# Small Bowel Intussusception in an Adult Male With a Large Jejunal Polyp as a Lead Point: A Rare Presentation

**DOI:** 10.7759/cureus.72909

**Published:** 2024-11-03

**Authors:** Ahmed Abdelkader, Antonio Golpe, Anupam Chandran, Ajay Dabra

**Affiliations:** 1 General Surgery, Scunthorpe General Hospital, Scunthorpe, GBR; 2 Upper Gastrointestinal Surgery, Scunthorpe General Hospital, Scunthorpe, GBR; 3 Radiology, Scunthorpe General Hospital, Scunthorpe, GBR

**Keywords:** intussusception in adults, jejunal intussusception, small-bowel obstruction, small bowel polyp, small bowel resection

## Abstract

Small bowel intussusception in adults is an uncommon presentation that could be initiated by small bowel polyps. This case report highlights an adult patient diagnosed with jejunojejunal intussusception caused by a large jejunal polyp as the lead point.

## Introduction

Intussusception occurs when a portion of the digestive system telescopes into a nearby portion ​[1]​. This uncommon condition accounts for less than 0.1% of adult hospital admissions ​[2]​ and affects only two to three individuals per 100,000 people annually ​[3,4]​.

Most individuals with intussusception present with symptoms of intestinal obstruction, often requiring emergency care. Diagnosis is typically achieved through radiological studies, such as CT scans with or without oral contrast, or during laparotomy. Diagnosing non-emergency cases can be challenging, as symptoms like intermittent abdominal pain frequently resolve on their own ​[5]​. When examinations and investigations do not reveal the condition, patients may be misdiagnosed with irritable bowel syndrome (IBS) or biliary colic.

## Case presentation

**Figure 1:**
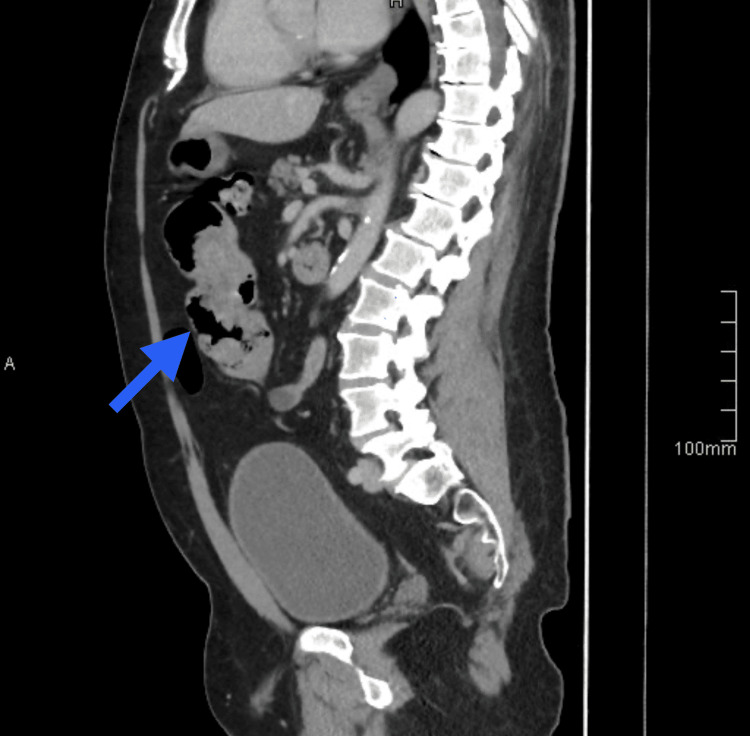
CT scan, sagittal view The arrow points to the intussuscepted segment.

**Figure 2:**
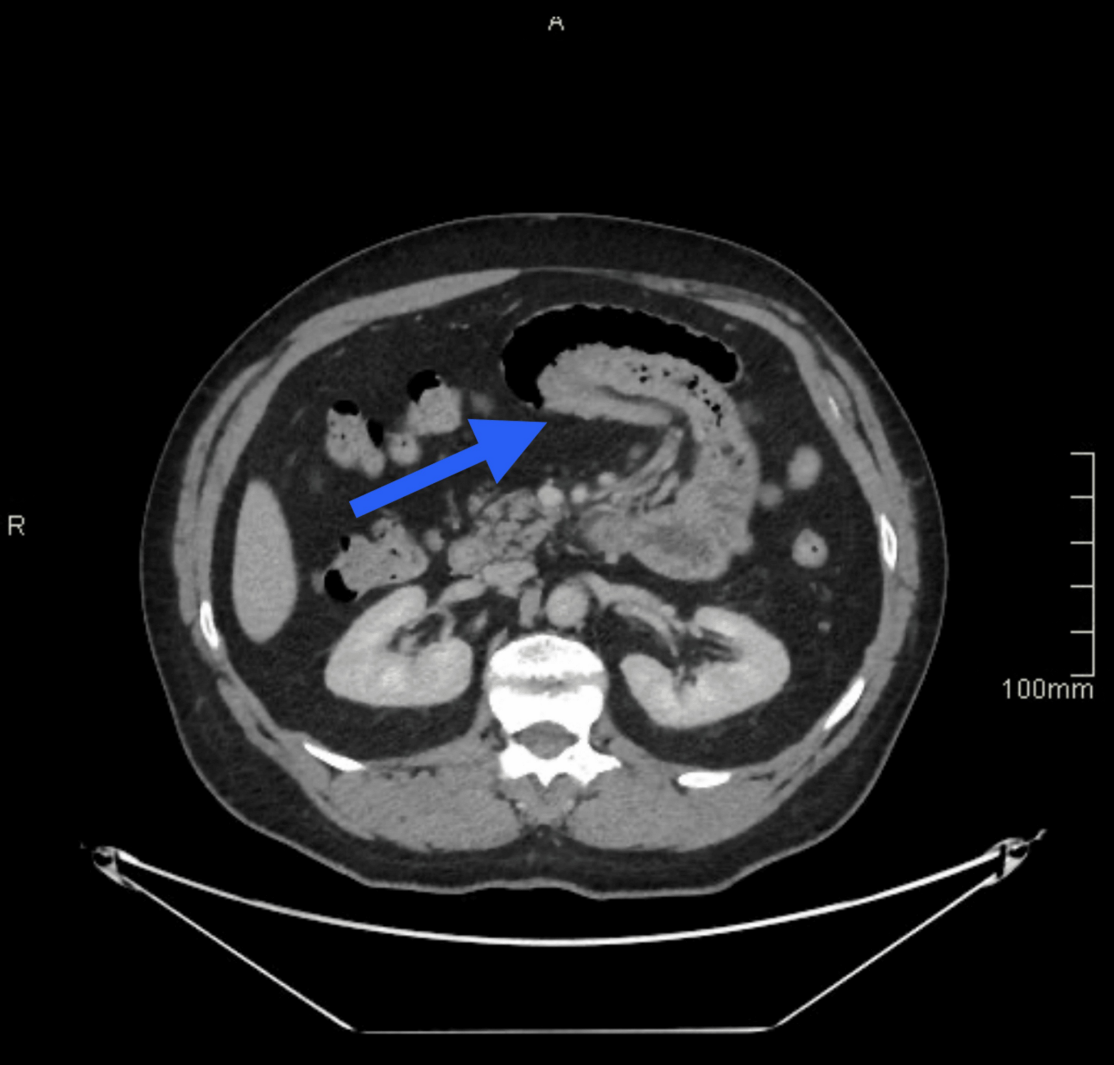
CT scan, axial view The arrow points to the intussuscepted segment.

We present a case of a 59-year-old male who arrived at the emergency department with severe abdominal pain and belching. Additionally, he reported a two-day history of anorexia and four days of constipation. His medical history included asthma, hypertension, benign prostatic hyperplasia (BPH), and pancreatic insufficiency. Laboratory investigations showed an elevated white blood cell count of 14 × 10^9^/L and a C-reactive protein (CRP) level of 150 mg/L, along with mild normal anion gap metabolic acidosis. Initially, his symptoms were suspected to be gallbladder-related; however, an abdominal ultrasound showed no gallstones or inflammation. A subsequent contrast-enhanced CT scan revealed jejunal intussusception without small bowel obstruction, as well as another distal segment with possible less severe telescoping (Figures 1, 2). The lead point could not be visualized or excluded on the CT scan.

**Figure 3:**
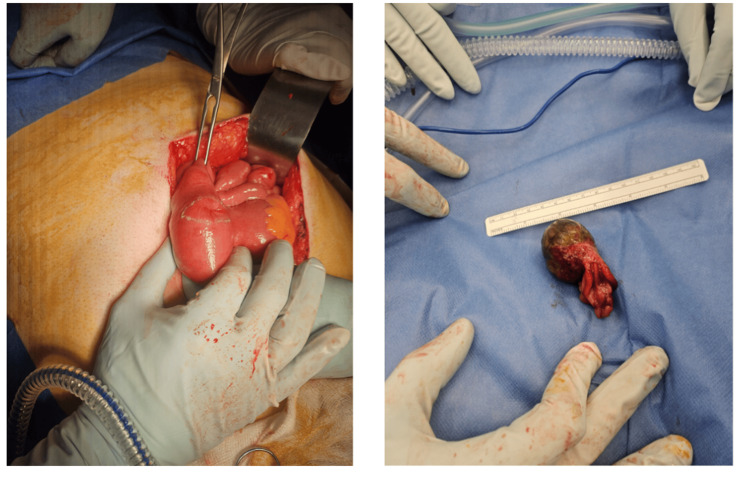
Intraoperative findings Photographs showing jejunal polyp as the lead point.

After senior review, an exploratory laparotomy was recommended for diagnosis and symptom management. A midline incision was made, and the intussuscepted jejunal segment was identified approximately 20 cm from the duodenojejunal (DJ) flexure, with 10 cm of the bowel length telescoped. No additional distal segments were telescoped as previously described on the CT scan. Reduction of the intussusception was performed, and a large 3 cm pedunculated polyp with a base at the antimesenteric border of the bowel was identified as the lead point (Figure 3). The decision was made to perform a wedge excision rather than bowel resection, as the polyp's base was narrow and the surrounding bowel appeared healthy. A wedge-shaped incision was made to excise the polyp, and the enterotomy was closed in two layers. Following surgery, the patient was transferred to the intensive care unit for monitoring before transitioning to the ward. He remained in the hospital for three days postoperatively and experienced an uneventful recovery. Histopathological examination of the specimen revealed features of leiomyoma with no evidence of malignancy.

It is worth noting that this patient had been under investigation for iron deficiency anemia for two years. Although he underwent both esophagogastroduodenoscopy (OGD) and colonoscopy, which were unremarkable, a capsule endoscopy was never conducted.

## Discussion

The majority of cases of intussusception occur in infants and children, with symptoms including abdominal pain, bloody stools, and the appearance of an abdominal mass ​[6]​. In adults, however, intussusception accounts for only about 5% of cases and is far less common. Adults typically present with less pronounced symptoms; the most common complaint is persistent abdominal pain, while bloody stools and palpable abdominal masses are less frequent ​[7]​. Additionally, over 90% of adult intussusception cases are secondary, usually due to underlying conditions like tumors or adhesions, whereas more than 90% of cases in children are primary ​[8]​. Recognizing these differences in presentation is crucial for timely diagnosis and treatment, especially in adults whose symptoms may be more subtle, leading to delays in medical attention.

In adults, intussusception is primarily caused by tumors, which account for 63-77% of cases. A significant proportion of tumor-related cases (50-73%) involve malignant tumors ​[9]​. Intestinal strictures caused by the tumor often lead to partial obstruction. Peristaltic waves may be disrupted at the tumor site, causing local irritation. Intussusception can result from several factors, including disruptions in peristaltic rhythm, stronger contractions, displacement of the tumor during peristalsis, or forward passage of intestinal contents ​[10]​. 

Surgical intervention is recommended upon diagnosis of adult intussusception. If there is no necrosis, a manual reduction should be performed, and the intestine thoroughly examined for tumors, polyps, diverticula, focal necrosis, and other abnormalities. If necrosis is present, immediate intestinal resection is required, and manual reduction is not advised. To prevent the potential spread of cancer cells, manual reduction should be avoided in cases where malignant tumors are suspected. Instead, lymph node dissection and intestinal resection should be performed, with direct anastomosis of the small intestine once the lesion is removed [11].

## Conclusions

Adult intussusception is rare and presents with non-specific, sporadic symptoms, making diagnosis challenging. Identifying the condition requires a high degree of clinical suspicion. Abdominal CT scans are the most effective diagnostic tool. If there is suspicion of cancer or non-viable tissue in the small bowel, the affected region should be surgically removed without attempting to reduce the lesion, unlike in colonic cases.
